# Yearling laryngeal function in Thoroughbreds that underwent a laryngoplasty differs from controls

**DOI:** 10.1111/evj.14110

**Published:** 2024-06-07

**Authors:** Josephine L. Hardwick, Benjamin J. Ahern, Kylie L. Crawford, Kate J. Allen, Samantha H. Franklin

**Affiliations:** ^1^ School of Animal and Veterinary Sciences University of Adelaide Roseworthy South Australia Australia; ^2^ School of Veterinary Medicine Murdoch University Murdoch Western Australia Australia; ^3^ School of Veterinary Science University of Queensland Gatton Queensland Australia; ^4^ Mater Research Institute University of Queensland Brisbane Queensland Australia; ^5^ School of Veterinary Sciences University of Bristol Bristol UK

**Keywords:** horse, larynx, sale, surgery, upper airway

## Abstract

**Background:**

Yearling laryngeal function (YLF) is frequently assessed at the time of sale and the outcomes of these assessments can have significant economic implications. The YLF of horses that subsequently underwent a prosthetic laryngoplasty (PL) is unknown.

**Objectives:**

We hypothesised horses with YLF ≥grade II.2 would be at increased risk of requiring PL, compared with YLF <grade II.2.

**Study design:**

Case–control.

**Methods:**

There were 150 PL cases from 2019 to 2021 with an available yearling post‐sale videoendoscopic examination and 600 controls. Two observers unaware of the outcome graded YLF using the Havemeyer system. The risk of PL for each YLF grade was calculated using multivariable conditional logistic regression.

**Results:**

The proportions of each YLF grade in the control group and PL group, respectively, were grade I: 25.8% and 13.3%, grade II.1: 54.3% and 35.3%, grade II.2: 16.7% and 26%, grade III.1: 3% and 20.7%, grade III.2: 0.2% and 3.3%, grade III.3: 0% and 0.7%, grade IV: 0% and 0.7%. The odds ratio (OR, 95% confidence interval) of requiring PL compared with the referent grade I were: grade II.1: 1.2 (0.7, 2.2, *p* = 0.5), grade II.2: 3.4 (1.8, 6.1, *p* < 0.001), grade III.1: 13.8 (6.0, 31.6, *p* < 0.001), grade III.2: 55.5 (10.3, 299.2, *p* < 0.001), grade III.3: 2930,000 (398173.7, 21 600,000, *p* < 0.001), grade IV: 26300,000 (3 420 000, 202 000 000, *p* < 0.001). Yearling LF ≥grade II.2 had an OR of 4.61 (3.0, 7.1, *p* < 0.001) compared with <grade II.2; YLF ≥grade III.1 had an OR of 10.7 (5.6, 20.4, *p* < 0.001) compared with <grade III.1.

**Main limitations:**

Lack of performance data to compare the PL and control groups. The control group was not ‘disease‐free’ and may have developed disease and been retired or undergone surgery elsewhere.

**Conclusions:**

Three‐quarters of the PL group had ≤grade II.2 YLF, demonstrating deterioration in LF post‐sale was common. The risk of requiring PL increased from YLF grade II.2 upwards.

## 总结

**背景:**

一岁马驹喉功能(YLF)经常在出售时进行评估，这些评估的结果可能具有重要的经济意义。接受喉修补成形术的马驹的YLF以往是未知的。.

**目的:**

我们假设:一岁马驹喉功能YLF≥II.2级的马驹，需要喉修补成形术PL的风险增加。.

**研究设计:**

1:4病例对照研究.

**方法:**

对2019‐2021年的150例PL病例的周岁拍卖内窥镜检查结果，和600例对照。双盲法Havemeyer系统对YLF进行评分。使用多变量条件逻辑回归计算每个YLF等级的PL风险。.

**结果:**

对照组和PL组各YLF分级所占比例分别为:I级:25.8%和13.3%； II.1级:54.3%和35.3%； II.2级:16.7%和26%；III.1级:1.3%和20.7%；III.2级:0.2%和3.3%；III.3级:0%和0.7%；IV级:0%和0.7%。.

与I级参考相比，需要PL的比值(OR, 95%置信区间)为: II.1级:1.2 (0.7，2.2，*p* = 0.5)； ii.2级:3.4 (1.8，6.1，*p* < 0.001)， iii.1级:13.8 (6.0，31.6，*p* < 0.001)， iii.2级:55.5 (10.3，299.2，*p* < 0.001)； iii.3级:2930000 (398173.7，21 600 000，*p* < 0.001)， IV级:26300000 (3 420 000,202 000 000，*p* < 0.001)。与LF小于II.2级的马驹相比，LF≥II.2级的OR为4.61 (3.0，7.1，*p* < 0.001)；与 < III.1级相比，YLF≥III.1级的OR为10.7 (5.6，20.4，*p* < 0.001)。.

**主要限制:**

缺乏其他性能数据来比较试验组和对照组。对照组并非“无病”，可能也患病，退役或有其他位置手术。.

**结论:**

PL组3 / 4患者的YLF≤II.2级，表明马驹拍卖后喉功能恶化较为常见。需要PL的风险从YLF II.2级上升。.

## 1 INTRODUCTION

Recurrent laryngeal neuropathy (RLN) is a degenerative and highly prevalent condition that causes neurogenic atrophy and dysfunction of the intrinsic laryngeal muscles.^1^, ^2^, ^3^ The true prevalence of RLN is difficult to ascertain because there is no universally accepted definition of the disease, but between 2.6% and 8.3% of horses are reported to be clinically affected by RLN,^4^ and histological evidence of neuromuscular changes were identified in 18.4%–41.6% of clinically unaffected horses.^2^, ^3^, ^4^, ^5^ In severe cases, left arytenoid cartilage abduction is unable to be maintained during exercise, leading to inspiratory stridor and exercise intolerance.^6^, ^7^ The onset of clinical signs in racing Thoroughbreds typically occurs after they have commenced race training, around 2–3 years of age.^8^, ^9^ This combination of factors makes laryngeal function assessment in asymptomatic yearlings a diagnostic challenge, which is further heightened by the economic implications of these assessments.^10^

Various treatment options are available, but all offer imperfect surgical solutions.^11^, ^12^, ^13^, ^14^, ^15^ Currently, prosthetic laryngoplasty (PL) is the most common surgical treatment for RLN in the racehorse, involving the placement of a prosthesis between the cricoid cartilage and muscular process of the left arytenoid cartilage to permanently stabilise the arytenoid cartilage and prevent collapse during exercise.^8^ The success rate in racehorses following PL varies between 50% and 70% depending on the outcome used to define success.^12^, ^13^, ^16^, ^17^, ^18^, ^19^

The laryngeal function of Thoroughbred yearlings presented at sale is frequently assessed via resting endoscopic examination and the outcomes of these assessments can have significant economic implications on the Thoroughbred industry.^10^, ^20^, ^21^, ^22^, ^23^ In Australia, laryngeal function at yearling sales is evaluated using a 5‐point grading system,^24^ whereas a 7‐point grading system is used in North America.^25^ Yearling laryngeal function (YLF) grade has been shown to be useful at predicting future performance for horses with normal laryngeal function (grades I and II.1) and abnormal laryngeal function (≥grade III.2) but the future performance of horses with intermediate laryngeal function (grades II.2 and III.1)^25^ is less clear.^20^, ^21^, ^23^, ^26^ Additionally, there is minimal data available on the YLF in horses that subsequently developed clinical signs of RLN requiring surgical treatment. In Australia, yearlings with grade 3 out of 5 laryngeal functions (equivalent to grade II.2 and III.1 on the 7‐point scale) currently pass the conditions of sale,^27^ but they are undesirable to purchasers. This has led to a widespread uptake in pre‐sales endoscopy, resulting in reduced sales prices and clearance rates for yearlings identified with grade 3 out of 5 laryngeal function pre‐sale.^10^, ^22^, ^23^

The aim of this study was to compare the YLF grades of horses that required a PL, to those of a control group. We hypothesised that horses with a YLF ≥grade II.2 would be at increased risk of requiring PL, compared with those with a YLF grade <II.2.

## 2 MATERIALS AND METHODS

### 2.1 Study design

This was a case–control study with a 1:4 ratio, where the outcome was a PL (case) or unknown outcome (control) and the exposure was YLF grade.

### 2.2 Inclusion criteria

Surgery records and yearling post‐sale videoendoscopic examinations were obtained from six large Australian equine hospitals. Horses were included in the PL group if they underwent a left‐sided PL between 2019 and 2021 (inclusive) and a yearling post‐sale videoendoscopic examination was available. For each horse in the PL group, four controls were chosen randomly from the same sale, providing that a post‐sale videoendoscopic examination was available and there was no known history of any upper airway surgery (from available surgery records and online race records). Despite this, the term ‘unknown outcome’ is used to describe the control group, as full medical records for all horses were not available and horses may have been retired or undergone surgery elsewhere. Recurrent laryngeal neuropathy can also be a subclinical disease, so it is difficult to classify controls as ‘disease‐free’.^28^ Horse sex, yearling sale, year of sale and the sales price were recorded.

### 2.3 Grading of videoendoscopic examinations

Two observers experienced in grading laryngeal function and unaware of the outcome (PL or unknown outcome) graded each video. Additionally, 50 videos were graded twice by each observer, to allow intra‐observer agreement to be assessed. The following observations were recorded: laryngeal function grade using the 7‐point grading system adapted into a diagnostic decision tree (see below); intermittent dorsal displacement of the soft palate (DDSP, yes/no); epiglottic grade (0–4)^29^; and the presence of ventro‐medial luxation of the apex of the corniculate process of the arytenoid cartilage (VLAC, left under right/right under left/bilateral/no).^30^, ^31^

If both observers did not agree on the laryngeal function grade, a third observer, who was also unaware of the outcome, provided a consensus grade for use in further analysis.

Due to previously identified disagreement between veterinarians when grading laryngeal function, a novel diagnostic decision tree, adapted from the 7‐point grading system, was used when grading YLF.^10^, ^25^ This decision tree aims to assist with an accurate categorisation of YLF and consists of a series of dichotomous (yes/no) decisions, that lead the observer down a pathway toward the most appropriate laryngeal function grade. The diagnostic decision tree was developed to clearly define certain parameters described in the 7‐point grading system (i.e., the definition of maintenance of arytenoid cartilage abduction) and aims to encourage observers to interpret the grading system similarly. The diagnostic decision tree is displayed in Figure S1.

### 2.4 Statistical analyses

#### 2.4.1 Power calculations

Power calculations were based on the findings of Barakzai and Dixon, whereby the probability of horses with a resting laryngeal function grade III (including all subgrades) having grade A (normal) exercising function on treadmill endoscopy was 34%.^32^ Thus, the probability of the exposure (resting laryngeal function grade III.1 and above) in the control group was 34%, with the inherent assumption that grade III resting laryngeal function/grade A exercising laryngeal function would not require prosthetic laryngoplasty. Using an asymptotic *z*‐test with a 1:4 matched design, we determined that 83 cases would be required to detect an odds ratio of 2.0, at a power of 80%.

#### 2.4.2 Data analysis

Statistical analyses were performed using Stata 18® (Statacorp, College Station, TX). The normality of continuous data was assessed using histograms. Non‐normally distributed data were presented as median, interquartile range. Categorical data were presented as numbers and percentages. Demographic and endoscopic characteristics we compared using two‐sample Wilcoxon rank‐sum tests, Pearson chi‐squared tests or Kruskal–Wallis ANOVA, according to data type.

The effect of laryngeal grade on whether horses had a PL or unknown outcome was assessed using conditional logistic regression and results are displayed as the odds ratio (OR) and 95% confidence interval (95% CI). This method of analysis corrects for misspecification due to any differences arising from the sale of origin. The assumption of linearity between continuous variables and the log odds of PL was checked by generating the predicted probabilities and categorising them into four equal categories. The continuous variable was then divided into four categories and the odds of PL for each category were determined. The mean value of the predicted probability for each category was plotted against the odds of PL for each category. If the association was not linear, the variable was analysed as a categorical variable.

**FIGURE 1:**
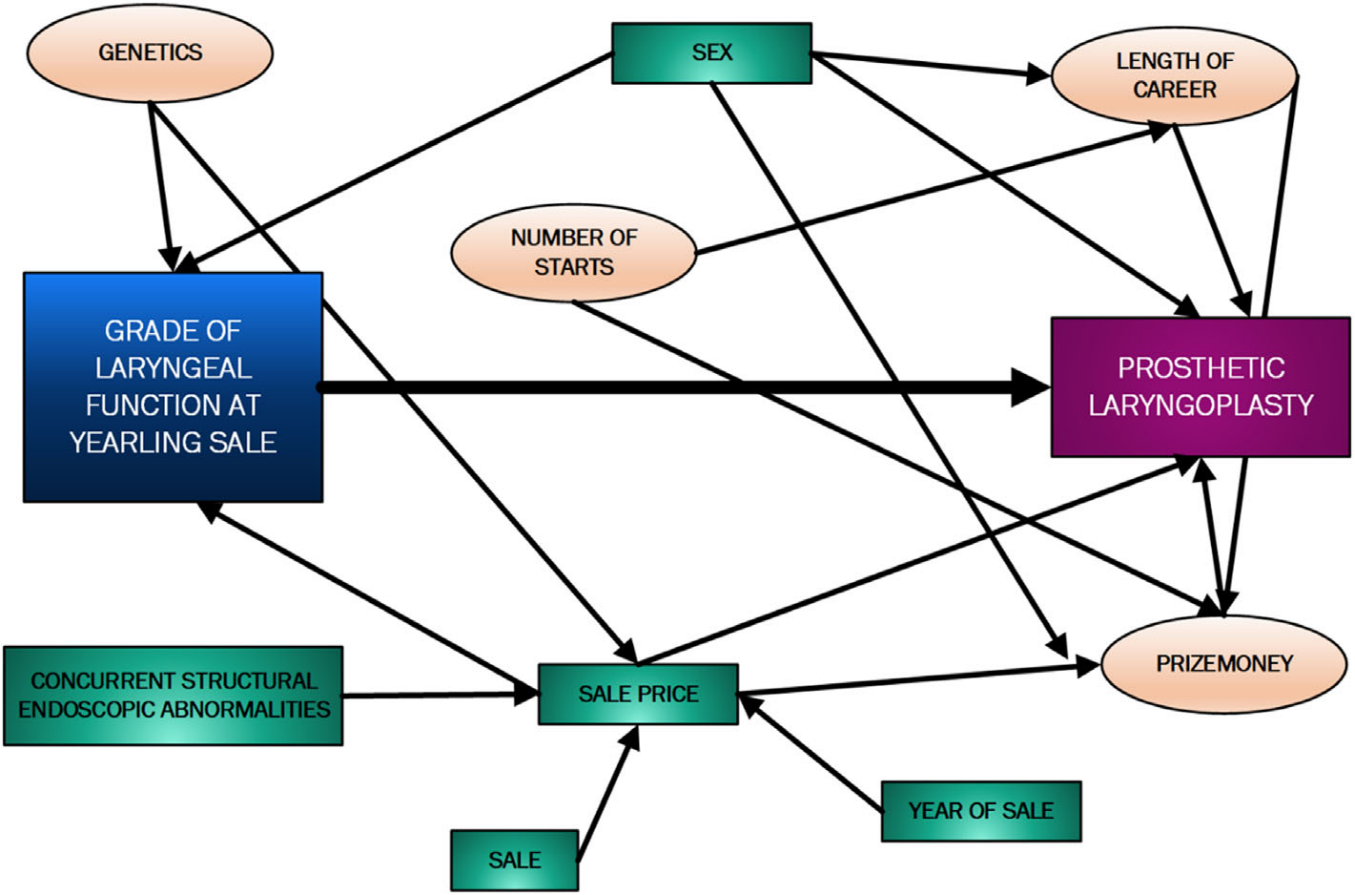
A directed acyclic graph depicting the causal inter‐relationship of variables affecting the grade of yearling laryngeal function and whether a prosthetic laryngoplasty was performed. Green boxes indicate measured variables and orange ovals represent unmeasured variables.

Univariable conditional logistic regression was performed with laryngeal function classified according to the 7‐point grading system. Multivariable conditional logistic regression models were built with variables considered for inclusion based on clinical relevance (Figure 1). In other words, for a confounder to be included it had to be causally associated with the exposure and outcome. Subsequent models were built with laryngeal function classified as grade II.2 and above and grade III.1 and above.

#### 2.4.3 Inter‐rater and intra‐rater agreement and reliability

The frequencies of assigned laryngeal grades and the percentages of agreement between observers were calculated. Cohen Kappa statistic of reliability was then calculated, using a quadratic weighting to account for the non‐linear difference in severity between differences in functional grade. Analyses were repeated comparing assigned grades for repeated observations for observers 1 and 2.

## 3 RESULTS

### 3.1 Recruitment data

**FIGURE 2:**
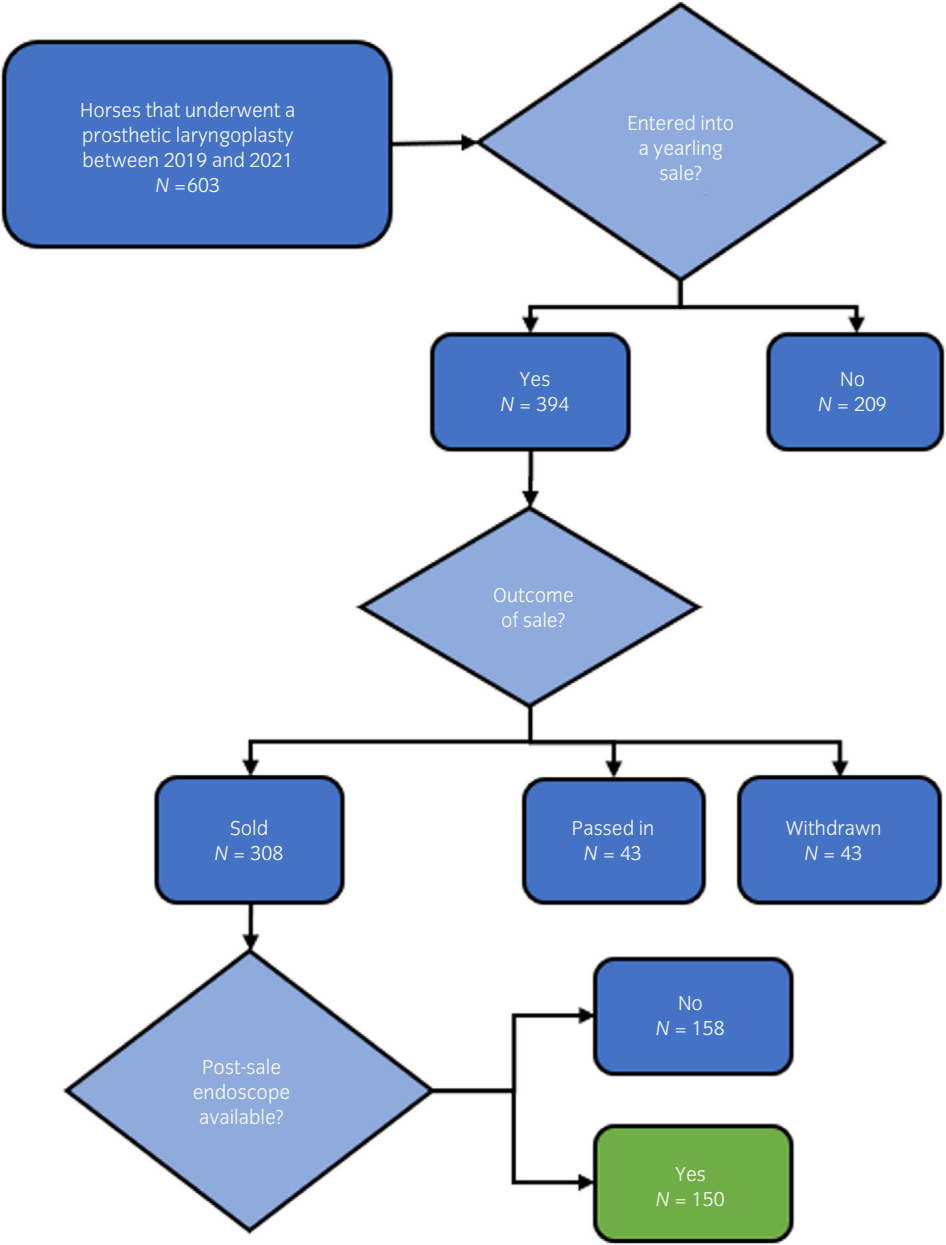
Flowchart depicting the recruitment data for horses in the prosthetic laryngoplasty group.

Out of the 603 horses that underwent a PL during the study period, only 150 had a post‐sale endoscopic exam available and were eligible for inclusion in the study. Recruitment data for the PL group are displayed in Figure 2. Four controls were selected from the same sale as each PL case, therefore there were 600 horses in the unknown outcome group.

### 3.2 Demographic data

Horse sex, yearling sale, year of sale, sales price, and yearling endoscopic findings (excluding YLF grade) stratified by outcome are displayed in Table 1. At time of PL surgery, the median horse age was 3 years (IQR 3, 4): 26 horses were 2‐year‐olds, 70 horses were 3‐year‐olds, 41 were 4‐year‐olds, 9 were 5‐year‐olds, 3 were 6‐year‐olds and 1 horse was 7 years old. There was no significant difference between the two groups in terms of yearling sale, year of sale, epiglottic grade and presence of VLAC. The percentage of males in the PL group (*n* = 117, 78%) was higher than in the unknown outcome group (*n* = 339, 56.5%, *p* < 0.001). The median sales price was higher in the PL group (AUD 110 000, IQR $50 000, 180 000) compared with the unknown outcome group (AUD 82 500, IQR $35 000, $170 000, *p* = 0.03). Dorsal displacement of the soft palate was observed more frequently in the PL group (42.7%) compared with the unknown outcome group (26.5%, *p* < 0.001).

**TABLE 1:** Demographic data and yearling endoscopic findings stratified by outcome (prosthetic laryngoplasty vs. unknown outcome).

	Total	Horses with unknown outcome	Horses with prosthetic laryngoplasty	*p*‐value
	*N* = 750	*N* = 600	*N* = 150	
Sex				<0.001
Female	294 (39.2%)	261 (43.5%)	33 (22.0%)	
Male	456 (60.8%)	339 (56.5%)	117 (78.0%)	
Sale				1.00
1	60 (8.0%)	48 (8.0%)	12 (8.0%)	
2	110 (14.7%)	88 (14.7%)	22 (14.7%)	
3	30 (4.0%)	24 (4.0%)	6 (4.0%)	
4	80 (10.7%)	64 (10.7%)	16 (10.7%)	
5	5 (0.7%)	4 (0.7%)	1 (0.7%)	
6	5 (0.7%)	4 (0.7%)	1 (0.7%)	
7	345 (46.0%)	276 (46.0%)	69 (46.0%)	
8	10 (1.3%)	8 (1.3%)	2 (1.3%)	
9	10 (1.3%)	8 (1.3%)	2 (1.3%)	
10	30 (4.0%)	24 (4.0%)	6 (4.0%)	
11	65 (8.7%)	52 (8.7%)	13 (8.7%)	
Year of sale				0.3
2014	5 (0.7%)	4 (0.7%)	1 (0.7%)	
2015	13 (1.7%)	13 (2.2%)	0 (0.0%)	
2016	41 (5.5%)	34 (5.7%)	7 (4.7%)	
2017	166 (22.1%)	135 (22.5%)	31 (20.7%)	
2018	183 (24.4%)	140 (23.3%)	43 (28.7%)	
2019	267 (35.6%)	209 (34.8%)	58 (38.7%)	
2020	75 (10.0%)	65 (10.8%)	10 (6.7%)	
Sale price (AUD)	90 000 (40 000, 170 000)	82 500 (35 000, 170 000)	110 000 (50 000, 180 000)	0.03
Epiglottic grade^a^				0.8
0	33 (4.4%)	27 (4.5%)	6 (4.0%)	
1	303 (40.4%)	249 (41.5%)	54 (36.0%)	
2	329 (43.9%)	258 (43.0%)	71 (47.3%)	
3	81 (10.8%)	63 (10.5%)	18 (12.0%)	
4	4 (0.5%)	3 (0.5%)	1 (0.7%)	
DDSP^b^	223 (29.7%)	159 (26.5%)	64 (42.7%)	<0.001
VLAC^c^				0.5
No	739 (98.8%)	591 (98.8%)	148 (98.7%)	
Left under right	7 (0.9%)	6 (1.0%)	1 (0.7%)	
Right under left	2 (0.3%)	1 (0.2%)	1 (0.7%)	
Concurrent endoscopic abnormality^d^	85 (11.3%)	66 (11.0%)	19 (12.7%)	0.6

*Note*: Data are presented as median (IQR) for continuous measures, and *n* (%) for categorical measures.

^a^ As defined by Pierce and Embertson.^29^

^b^ DDSP = dorsal displacement of the soft palate.

^c^ VLAC = ventromedial luxation of the apex of the corniculate process of the arytenoid.^30^

^d^ Defined as including ≥1 of the following: epiglottic grade ≥3; DDSP; presence of VLAC.

Demographic data and yearling endoscopic findings stratified according to YLF grade are displayed in Table S1. Horse sex, year of sale, sales price, epiglottic grade and presence of VLAC were not significantly different between YLF grades. Yearling sale and the frequency of DDSP were different between laryngeal function grades (*p* = 0.02 and *p* < 0.001, respectively).

### 3.3 Laryngeal function grades

**FIGURE 3:**
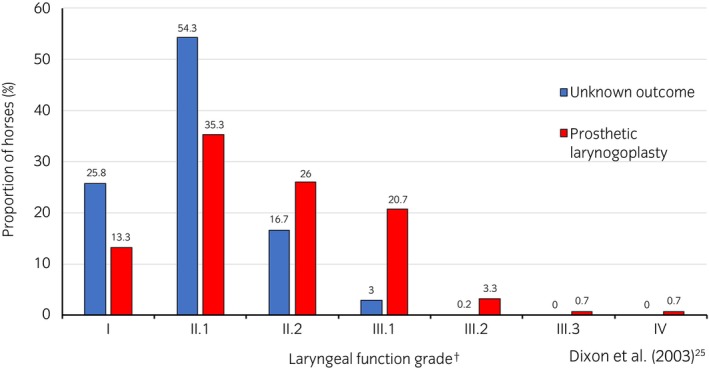
Bar chart depicting the proportion of Thoroughbred yearling laryngeal function grades in the prosthetic laryngoplasty group compared with those of unknown outcome.

The proportion of horses with each laryngeal function grade in both the PL group and the unknown outcome group is displayed in Figure 3. The frequencies of each YLF grade across both groups are displayed in Table 2.

**TABLE 2:** Yearling laryngeal function grade frequencies and column percentages for horses in both the unknown outcome group and prosthetic laryngoplasty group.

Outcome	Yearling laryngeal function grade^a^							
	I	II.1	II.2	III.1	III.2	III.3	IV	Total
Unknown	155	327	99	18	1	0	0	600
	88.6	86.1	71.7	36.7	16.7	0	0	80
Laryngoplasty	20	53	39	31	5	1	1	150
	11.4	14.0	28.3	63.3	83.3	100	100	20
Total	175	380	138	49	6	1	1	750
	100	100	100	100	100	100	100	100

*Note*: First row listed for each group displays *frequencies*; second row displays *column percentages*.

^a^ Dixon et al.^25^

### 3.4 Multivariable conditional logistic regression

Model results on the effect of YLF grade on outcome are displayed in Table 3. Univariable results are displayed in Table S2. The adjusted OR (95% CI) between yearlings with grade I and grade II.1 laryngeal function was 1.2 (0.7, 2.2) and was not statistically significant (*p* = 0.5). The adjusted OR (95% CI) of yearlings with laryngeal function grade II.2 requiring PL compared with yearlings with grade I laryngeal function was 3.3 (1.8, 6.1). Yearling laryngeal function grades above grade II.2 had higher odds of requiring surgery.

**TABLE 3:** Multivariable conditional logistic regression model of the effect of laryngeal function grade on whether the horse had a prosthetic laryngoplasty or unknown outcome.

Laryngeal function grade^a^	Multivariable OR^b^ (95% CI)	*p*‐value
I (referent)	1.00	
II.1	1.2 (0.7, 2.2)	0.5
II.2	3.3 (1.8, 6.1)	<0.001
III.1	13.8 (6.0, 31.6)	<0.001
III.2	55.5 (10.3, 299.2)	<0.001
III.3	2 930 000 (398 173.7, 21 600 000)	<0.001
IV	26 300 000 (3 420 000, 202 000 000)	<0.001
Below Grade II.2 (referent)	1.00	
Grade II.2 and above	4.6 (3.0, 7.1)	<0.001
Below Grade III.1 (referent)	1.00	
Grade III.1 and above	10.7 (5.57, 20.4)	<0.001

Abbreviations: CI, confidence interval; OR, odds ratio.

^a^ Dixon et al.^25^

^b^ Adjusted for sex and sale price.

### 3.5 Percentage of agreement

Observer 1 and observer 2 assigned identical YLF grades to the same endoscopic video in 454 horses (60.5%), and they differed by 1 grade in 295 horses (39.3%) and by 2 grades for 1 horse (0.1%). The most frequent disagreements were between grades I and II.1 in 184 horses (62.2% of the differences). The remainder of the disagreements were mainly between grades II.2 and III.1 (85 horses, 28.7% of differences) and grades III.1 and III.2 (24 horses, 8.1% of differences). The observers disagreed once between grades III.2 and III.3, grades III.3 and grade IV and grades III.2 and II.2.

### 3.6 Cohen kappa measure of reliability

A quadratic weighting between laryngeal function grades was applied, providing an overall level of agreement between observer 1 and 2 of 98.9%. The Cohen's Kappa statistic of reliability was also high (Kappa = 0.77; 95% CI 0.7, 0.8).

## 4 DISCUSSION

A major finding of this study was that almost half of the horses in the PL group had ‘normal’ YLF (grades I and II.1) and a further 26% had an equivocal grade (grade II.2) at the time of sale. In a previous study, 93% of mixed breed mature horses with grade I–II.2 resting laryngeal function had normal (grade A) exercising function.^32^ Additionally, PL is invariably performed on horses with grade III.1 laryngeal function and above,^33^, ^34^ therefore the laryngeal function of most horses in the surgery group must have deteriorated. Progression of RLN has also been identified in 15% of adult horses examined over a median period of 12 months,^35^ and 12% of young Thoroughbreds over a period of 16‐months or more.^36^ Our results indicate the importance of educating buyers about the potential for RLN to progress, even in yearlings with laryngeal function grades I or II.1.

The aetiology of RLN remains elusive but it is likely multifactorial and related to extreme nerve length.^28^ Given the progressive nature of RLN and the high prevalence of subclinical disease, it may be better to view the condition as a pathological process on a continuum rather than as a discrete entity.^2^, ^3^, ^4^, ^5^ In other words, assuming resting laryngeal function grade accurately correlates horses with the appropriate degree of RLN,^37^ yearlings should not be categorised into either an affected group or a disease‐free group, but instead considered to exist at different points along the disease pathway with different odds of requiring a PL. A risk‐based assessment may therefore be more appropriate when examining YLF at sales, but this needs to be validated with performance data.

There was no significant difference in the odds of requiring surgery between yearlings with grade I and grade II.1 laryngeal function. Coupled with the results of other studies that found no difference between these grades in terms of future performance,^21^, ^23^ or arytenoid cartilage grade at exercise,^32^ it appears that categorising grades I and II.1 as low risk of developing clinically significant future laryngeal function is reasonable.

The significant increase in the odds of requiring a PL with each grade of laryngeal function from grade II.2 and upwards supports the use of the 7‐point grading system, or a simplified risk‐based assessment at yearling sales.^23^ As expected, yearlings with significant arytenoid abductor deficit (grade III.2 and above) had more than 55 times the odds of requiring a PL compared with yearlings with normal (grade I) laryngeal function. Similarly, Barakzai and Dixon found 88.5% of mature horses that presented with a history of poor performance and/or abnormal respiratory noise, with grade III.2 or above resting laryngeal function, had arytenoid cartilage collapse (grade B or C) during exercise.^32^ Therefore grades III.2 and above should be considered at high risk of future laryngeal dysfunction. However, the interpretation of the risk category for yearlings with grade II.2 and III.1 laryngeal function is not as clear.

We chose to use yearling post‐sale endoscopic exams, rather than pre‐sale exams, because they are useful at predicting performance in previous studies.^20^, ^21^, ^23^ Additionally, as post‐sale exams are performed by independent, sales‐nominated veterinarians, the potential for vendor bias, including alterations in endoscopic technique to achieve a favourable result, should be minimised. A comparison of yearling pre‐sale versus post‐sale endoscopic findings is warranted.

We graded yearling laryngeal function with the 7‐point system, rather than the 5‐point system used at Australian yearling sales, because it allows horses with prolonged rima glottidis asymmetry to be separated into those that can achieve and maintain full arytenoid cartilage abduction (grade II.2), from those that can achieve but not maintain full abduction (grade III.1). Yearlings with grade III.1 function had significantly higher odds of requiring a PL, compared with yearlings with grade II.2 function, demonstrating the strength of the 7‐point system for use in assessment of YLF. Similarly, another study identified horses with grade II.2 YLF outperformed horses with grade III YLF as a 3‐ and 4‐year‐old, but they earnt significantly less as a 4‐year‐old compared with horses with grade I and II.1 YLF.^21^ The aforementioned study did not separate horses with grade III YLF into subcategories due to study numbers, therefore comparisons between their findings and ours require caution. In contrast, a study using the 5‐point system identified no difference in performance between yearlings with grade 3 laryngeal function and those with normal laryngeal function.^26^

A comparison of resting and exercising laryngeal function in adult horses referred for poor performance found 44% of horses with grade III.1 resting laryngeal function had abnormal exercising function (grade B or C), compared with only 7% of horses with grade II laryngeal function and below.^32^ There is little data available comparing exercising laryngeal function between horses with resting laryngeal function grades II.2 and III.1; however, one study investigating transoesophageal ultrasound of the cricoarytenoideus dorsalis muscle (CADM), reported normal exercising function in 89% and 17% of horses with grade II.2 and grade III.1 laryngeal respectively.^38^ Our findings indicate there is a significant difference in risk of future laryngeal dysfunction between horses with grade II.2 and III.1 YLF; however, an appropriately powered study investigating race performance data is required to determine if this difference in risk translates into reduced earnings on the racetrack.

The use of additional diagnostic tests to further evaluate yearlings with grade II.2 and III.1 resting laryngeal function is warranted. A UK study found overground exercising endoscopy to be well tolerated in yearlings and identified abnormal exercising function in 3 out of 8 yearlings with grade II.2 or III.1 resting laryngeal function, whereas all yearlings with grade I or II.1 laryngeal function had normal exercising function.^39^ Laryngeal ultrasonography of the cricoarytenoideus lateralis muscle was shown to be more sensitive and specific at predicting arytenoid cartilage collapse during exercise in poorly performing adult racehorses compared with resting endoscopy,^40^ however it was unreliable at detecting subclinical RLN in clinically normal yearlings.^41^ Transcutaneous ultrasonography of the CADM may prove to be a useful tool in yearlings, but it has not been correlated with exercising endoscopic findings.^42^

Subclinical yearlings, deemed at increased risk of developing clinical RLN, would be ideal surgical candidates for newer CADM reinnervation and neuroprosthesis procedures, before development of progressive laryngeal muscle atrophy and fibrosis.^5^, ^15^, ^43^, ^44^, ^45^ Additionally, the required post‐operative convalescent time should not impact the onset of race training and early intervention should improve surgical outcomes. A modified C1/C2 nerve transplantation technique improved exercising laryngeal function grade in 8/14 horses with clinical signs of RLN, however we are not aware of any reports detailing the success rates of reinnervation techniques on subclinical cases of RLN.

Interestingly, there were more instances of DDSP in the PL group compared with the unknown outcome group. There is some clinical evidence that DDSP can co‐exist with RLN,^46^ however it was not considered to be a confounder in this study as it was not deemed to be causally associated with both the exposure and outcome. From our observations, increased duration of the endoscopic examination and increased laryngeal stimulation appeared to increase the frequency of DDSP, however no statistical analysis was performed between groups.

Despite the seemingly high proportion of horses that underwent PL being withdrawn (10.9%), or passed‐in (10.9%) at sale, the proportions were similar to the overall sales data collated from the four major select yearling sales in Australia between 2018 and 2020. The lack of sales data for approximately one‐third of horses (*n* = 209) that underwent PL may reflect farms not presenting to sale yearlings that were identified with higher laryngeal function grades pre‐sale, which was a sub‐theme identified from a series of focus groups with yearling stakeholders.^10^ Reasons for a lack of a post‐sale endoscopic examination in just over half of the yearlings sold at auction include incomplete access to all endoscopic exams during the time period, and purchasers opting out of a post‐sale exam.

The higher sales price in the PL group compared with the control group indicates owners may be more likely to operate on expensive horses to try and recoup their investment, and/or that yearlings with desirable genetics or phenotypic traits may be more likely to develop RLN (i.e., tall, well‐developed males).

The level of agreement when grading laryngeal function between the two experienced observers was higher than that reported by McLellan and Plevin in any observer group.^47^ This improved agreement may be explained by our use of a diagnostic decision tree to assist accurate categorisation of laryngeal function when using the 7‐point ordinal scale. Whilst the majority of interobserver disagreements were between YLF grades I and II.1, these are of minimal concern. However, disagreements between higher YLF grades have greater implications given the difference in risk of a PL between grades II.2 and above. In high stakes scenarios such as yearling sales, it may be more appropriate for yearlings to be assigned a consensus YLF grade by a panel of experienced veterinarians, rather than relying on a single observer. Future investigations should focus on more objective methods of evaluating YLF, such as computer‐assisted assessment. The development of training tools to assist veterinarians when grading laryngeal function is also warranted.

### 4.1 Limitations

The main limitation of the study is the lack of racehorse performance data to compare the control group and PL group, as this would determine the true relevance of grading YLF. Another potential limitation of this study is that the control group were not ‘disease‐free’, meaning they may have developed RLN and been retired, or undergone surgery but we were unaware of this outcome. Regardless, this limitation has the advantage of making our findings more robust, as it shifts them toward the null. In other words, it makes the observed effect size (odds ratio) smaller than the true effect size. A further limitation is the progressive nature of RLN, as discussed above. The potential for deterioration in laryngeal function is equally likely for cases and controls, thus this should not impact the robustness of our findings.

### 4.2 Conclusion

This study clearly demonstrates the probability of requiring a PL increases with YLF grade from grade II.2 and upwards. However, this study does not investigate performance or infer any effect on prize money earned before undergoing PL. The high prevalence of subclinical disease, coupled with the typical onset of clinical signs after purchase, increases the diagnostic challenge of assessing laryngeal function in asymptomatic yearlings at sale. The high proportion of horses with normal laryngeal function that subsequently underwent PL, indicates that classifying yearlings into different risk categories of laryngeal dysfunction may be an improved method of communicating the risks of clinical disease with clients.

## FUNDING INFORMATION

This study received no funding.

## CONFLICT OF INTEREST STATEMENT

The authors have declared no conflicting interests.

## AUTHOR CONTRIBUTIONS

**Josephine L. Hardwick:** Conceptualization; investigation; writing – original draft; project administration; methodology; formal analysis. **Benjamin J. Ahern:** Conceptualization; investigation; writing – review and editing; supervision; methodology. **Kylie L. Crawford:** Methodology; software; formal analysis; writing – review and editing; validation; supervision. **Kate J. Allen:** Supervision; writing – review and editing; validation; investigation. **Samantha H. Franklin:** Supervision; conceptualization; investigation; writing – review and editing; methodology.

## DATA INTEGRITY STATEMENT

Josephine L. Hardwick had full access to all the data in the study and takes responsibility for the integrity of the data and the accuracy of data analysis.

## ETHICAL ANIMAL RESEARCH

The University of Adelaide's animal ethics committee confirmed that ethical approval was not required for this retrospective study.

## INFORMED CONSENT

Owners/agents were aware that medical records may be used for research in general, but they were not aware of this explicit study.

### PEER REVIEW

The peer review history for this article is available at https://www.webofscience.com/api/gateway/wos/peer-review/10.1111/evj.14110.

## Supporting information


**Figure S1.** Diagnostic decision tree used to grade yearling laryngeal function.

**Table S1.** Demographic data and yearling endoscopic findings stratified by laryngeal function grade.

**Table S2.** Univariable conditional logistic regression model of the effect of laryngeal function grade on whether the horse had a prosthetic laryngoplasty or unknown outcome.

## Data Availability

The data that support the findings of this study are openly available in Figshare at https://figshare.com; reference number 25016678; doi: 10.6084/m9.figshare.25016678.
