# Supplementary figures and images for: Yearling laryngeal function in Thoroughbreds that underwent a laryngoplasty differs from controls
Source: Equine Vet J. 2024 Jun 7;57(2):431–40. doi: 10.1111/evj.14110 (PMC11807936; doi:10.1111/evj.14110)

**Figure S1.** Diagnostic decision tree used to grade yearling laryngeal function.

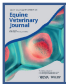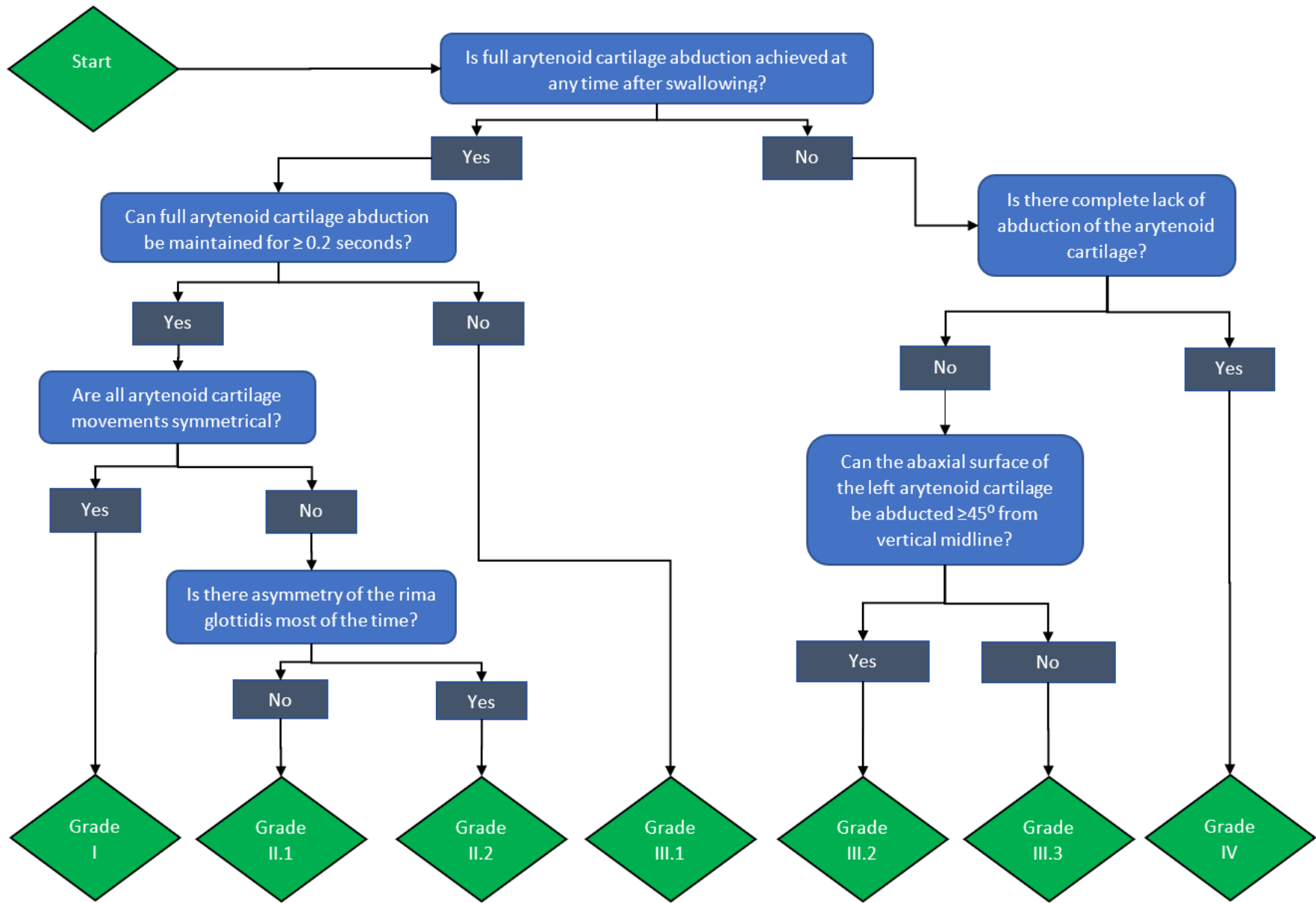

Supplement: Supplementary file 1 — Figure S1. Diagnostic decision tree used to grade yearling laryngeal function. [file EVJ-57-431-s002.pdf]
