# Supplementary material for: Yearling laryngeal function in Thoroughbreds that underwent a laryngoplasty differs from controls
Source: Equine Vet J. 2024 Jun 7;57(2):431–40. doi: 10.1111/evj.14110 (PMC11807936; doi:10.1111/evj.14110)
Supplement: Supplementary file 2 — Table S1. Demographic data and yearling endoscopic findings stratified by laryngeal function grade. [file EVJ-57-431-s003.pdf]

Equine Veterinary Journal BEVA

Table S1. Demographic data and yearling endoscopic findings stratified by laryngeal function grade.

		Laryngeal function grade[a]							
	Total	I	II.1	II.2	III.1	III.2	III.3	IV	p-value
	N=750	N=175	N=380	N=138	N=49	N=6	N=1	N=1	
**Sex**									0.6
Female	294 (39.2%)	77 (44.0%)	141 (37.1%)	57 (41.3%)	17 (34.7%)	2 (33.3%)	0 (0%)	0 (0%)	
Male	456 (60.8%)	98 (56.0%)	239 (62.9%)	81 (58.7%)	32 (65.3%)	4 (66.7%)	1 (100%)	1 (100%)	
**Sale**									0.02
1	60 (8.0%)	23 (13.1%)	28 (7.4%)	3 (2.2%)	5 (10.2%)	0 (0%)	0 (0%)	1 (100%)	
2	110 (14.7%)	29 (16.6%)	63 (16.6%)	10 (7.2%)	7 (14.3%)	0 (0%)	1 (100%)	0 (0%)	
3	30 (4.0%)	6 (3.4%)	17 (4.5%)	6 (4.3%)	1 (2.0%)	0 (0%)	0 (0%)	0 (0%)	
4	80 (10.7%)	11 (6.3%)	45 (11.8%)	18 (13.0%)	5 (10.2%)	1 (16.7%)	0 (0%)	0 (0%)	
5	5 (0.7%)	4 (2.3%)	1 (0.3%)	0 (0%)	0 (0%)	0 (0%)	0 (0%)	0 (0%)	
6	5 (0.7%)	1 (0.6%)	3 (0.8%)	1 (0.7%)	0 (0%)	0 (0%)	0 (0%)	0 (0%)	
7	345 (46.0%)	78 (44.6%)	174 (45.8%)	68 (49.3%)	23 (46.9%)	2 (33.3%)	0 (0.0%)	0 (0%)	
8	10 (1.3%)	3 (1.7%)	5 (1.3%)	2 (1.4%)	0 (0%)	0 (0%)	0 (0%)	0 (0%)	
9	10 (1.3%)	2 (1.1%)	5 (1.3%)	3 (2.2%)	0 (0%)	0 (0%)	0 (0%)	0 (0%)	
10	30 (4.0%)	10 (5.7%)	10 (2.6%)	6 (4.3%)	2 (4.1%)	2 (33.3%)	0 (0%)	0 (0%)	
11	65 (8.7%)	8 (4.6%)	29 (7.6%)	21 (15.2%)	6 (12.2%)	1 (16.7%)	0 (0%)	0 (0%)	
**Year of Sale**									0.3
2014	5 (0.7%)	4 (2.3%)	1 (0.3%)	0 (0%)	0 (0%)	0 (0%)	0 (0%)	0 (0%)	
2015	13 (1.7%)	3 (1.7%)	7 (1.8%)	3 (2.2%)	0 (0%)	0 (0%)	0 (0%)	0 (0%)	
2016	41 (5.5%)	8 (4.6%)	22 (5.8%)	11 (8.0%)	0 (0%)	0 (0%)	0 (0%)	0 (0%)	
2017	166 (22.1%)	30 (17.1%)	86 (22.6%)	40 (29.0%)	9 (18.4%)	1 (16.7%)	0 (0%)	0 (0%)	
2018	183 (24.4%)	45 (25.7%)	99 (26.1%)	23 (16.7%)	13 (26.5%)	2 (33.3%)	0 (0%)	1 (100%)	
2019	267 (35.6%)	57 (32.6%)	133 (35.0%)	52 (37.7%)	21 (42.9%)	3 (50.0%)	1 (100%)	0 (0%)	
2020	75 (10.0%)	28 (16.0%)	32 (8.4%)	9 (6.5%)	6 (12.2%)	0 (0%)	0 (0%)	0 (0%)	
**Sale Price AUD**	90000 (40000, 170000)	100000 (40000, 200000)	80000 (37250, 160000)	80000 (35000, 180000)	110000 (50000, 160000)	115000 (100000, 150000)	12000 (1200, 12000)	150000 (150000, 150000)	0.3
**Epiglottic grade**									0.5
0	33 (4.4%)	12 (6.9%)	12 (3.2%)	8 (5.8%)	1 (2.0%)	0 (0%)	0 (0%)	0 (0%)	
1	303 (40.4%)	74 (42.3%)	157 (41.3%)	47 (34.1%)	23 (46.9%)	2 (33.3%)	0 (0%)	0 (0%)	
2	329 (43.9%)	76 (43.4%)	169 (44.5%)	59 (42.8%)	21 (42.9%)	2 (33.3%)	1 (100%)	1 (100%)	
3	81 (10.8%)	12 (6.9%)	41 (10.8%)	23 (16.7%)	3 (6.1%)	2 (33.3%)	0 (0%)	0 (0%)	
4	4 (0.5%)	1 (0.6%)	1 (0.3%)	1 (0.7%)	1 (2.0%)	0 (0%)	0 (0%)	0 (0%)	
**DDSP[†]**	223 (29.7%)	38 (21.7%)	96 (25.3%)	60 (43.5%)	25 (51.0%)	3 (50.0%)	1 (100%)	0 (0%)	<0.001
**VLAC[‡]**									0.5
Not present	739 (98.8%)	173 (98.9%)	376 (99.5%)	135 (97.8%)	47 (95.9%)	6 (100%)	1 (100%)	1 (100%)	
Left under right	7 (0.9%)	2 (1.1%)	1 (0.3%)	3 (2.2%)	1 (2.0%)	0 (0%)	0 (0%)	0 (0%)	

**Right under left**	2 (0.3%)	0 (0.0%)	1 (0.3%)	0 (0.0%)	1 (2.0%)	0 (0%)	0 (0%)	0 (0%)	
**Concurrent Endoscopic Abnormality[§]**	85 (11.3%)	13 (7.4%)	42 (11.1%)	24 (17.4%)	4 (8.2%)	2 (33.3%)	0 (0%)	0 (0%)	0.08

Data are presented as median (IQR) for continuous measures, and n (%) for categorical measures.
[†] DDSP = Dorsal displacement of the soft palate, [‡] VLAC = Ventromedial luxation of the apex of the corniculate process of the arytenoid, [§] Defined as including ≥1 of the following: epiglottic grade ≥ 3; DDSP; presence of VLAC; [a] Dixon et al. [25].
