# Supplementary material for: Yearling laryngeal function in Thoroughbreds that underwent a laryngoplasty differs from controls
Source: Equine Vet J. 2024 Jun 7;57(2):431–40. doi: 10.1111/evj.14110 (PMC11807936; doi:10.1111/evj.14110)
Supplement: Supplementary file 3 — Table S2. Univariable conditional logistic regression model of the effect of laryngeal function grade on whether the horse had a prosthetic laryngoplasty or unknown outcome. [file EVJ-57-431-s001.pdf]

Equine Veterinary Journal BEVA

Table S2. Univariable conditional logistic regression model of the effect of laryngeal function grade on whether the horse had a prosthetic laryngoplasty or unknown outcome.

Laryngeal function grade†	Univariable OR (95% CI)	p-value
I (referent)	1.00	
II.1	1.3 (0.8, 2.3)	0.3
II.2	3.3 (1.8, 6.2)	<0.001
III.1	13.8 (6.1, 31)	<0.001
III.2	48.5 (6.9, 340.9)	<0.001
III.3	2520000 (344168.7, 18500000)	<0.001
IV	43500000 (5770000, 328000000)	<0.001
Below Grade II.2 (referent)	1.00	
Grade II.2 and above	4.3 (2.9, 6.4)	<0.001
Below Grade III.1 (referent)	1.00	
Grade III.1 and above	4.6 (3.0, 7.1)	<0.001

†Dixon et al. [25], OR = odds ratio, CI = confidence interval.
